# Supplementary material for: Structural basis of 3′-tRNA maturation by the human mitochondrial RNase Z complex
Source: EMBO J. 2024 Nov 8;43(24):15. doi: 10.1038/s44318-024-00297-w (PMC11649783; doi:10.1038/s44318-024-00297-w)
Supplement: Supplementary file 1 — Appendix [file 44318_2024_297_MOESM1_ESM.pdf]

Appendix

Structural basis of 3′-tRNA maturation by the human mitochondrial RNase Z complex

Genís Valentín Gesé[1] and B. Martin Hällberg[1,2]
1 Department of Cell and Molecular Biology, Karolinska Institutet, Solna, Sweden.
2 Centre for Structural Systems Biology (CSSB) and Karolinska Institutet VR-RÅC, Hamburg, Germany.

Table of contents

Appendix Table S1. 2
Appendix Figure S1 - Mitochondrial RNAse Z complex formation. 3
Appendix Figure S2 - Cryo-EM data-processing for the RNase Z-HS dataset. 5
Appendix Figure S3 - Resolution of the RNase Z-HS cryo-EM structure (EMD-50050). 6
Appendix Figure S4 - TRMT10C active site close-up 7
Appendix Figure S5 - Structure-based multiple sequence alignment of ELAC2 and TRMT10C homologs. 9
Appendix Figure S6 - Cryo-EM data-processing for the RNase Z^{H548A}-HS dataset. 11
Appendix Figure S7 - Resolution of the RNase Z^{H548A}-HS cryo-EM structure (EMD-50051). 12
Appendix Figure S8 - Cryo-EM data-processing for the RNase Z-HCCA dataset. 14
Appendix Figure S9 - Resolution of the RNase Z-HCCA cryo-EM structure (EMD-50052). 16
Appendix Figure S10 - Resolution of the RNase Z-HCCA cryo-EM structure (TRMT10C/SDR5C1 focus, EMD-51230). 18

Appendix Table S1.

Correspondence between nucleotide numbers in mitochondrial $tRNA^{His}$, which were used in the deposited structures, and in canonical tRNA, which were used throughout the manuscript.

tRNAHis	Canonical	Base	Region
1	1	G	Acceptor stem
2	2	U	
3	3	A	
4	4	A	
5	5	A	
6	6	U	
7	7	A	
8	8	A	
9	9	A	
10	10	G	D stem
11	11	U	
12	12	U	
13	13	U	
14	14	A	D loop
15	15	A	
16	16	C	
17	19	C	
18	20	A	
19	22	A	D stem
20	23	A	
21	24	A	
22	25	C	
23	26	A	
24	27	U	Anticodon stem
25	24	C	
26	25	A	
27	26	G	
28	31	A	
29	32	U	Anticodon loop
30	33	U	
31	34	G	
32	35	U	
33	36	G	
34	37	A	
35	38	A	
36	39	U	Anticodon stem
37	40	C	
38	41	U	
39	42	G	
40	43	A	
41	44	C	Variable region
42	45	A	
43	47	A	
44	48	C	
45	49	A	T stem
46	50	G	
47	51	A	
48	52	G	
49	53	G	
50	54	C	T loop
51	55	U	
52	56	U	
53	57	A	
54	58	C	
55	59	G	
56	60	A	
57	61	C	T stem
58	62	C	
59	63	C	
60	64	C	
61	65	U	
62	66	U	Acceptor stem
63	67	A	
64	68	U	
65	69	U	
66	70	U	
67	71	A	
68	72	C	
69	73	C	Discriminator base

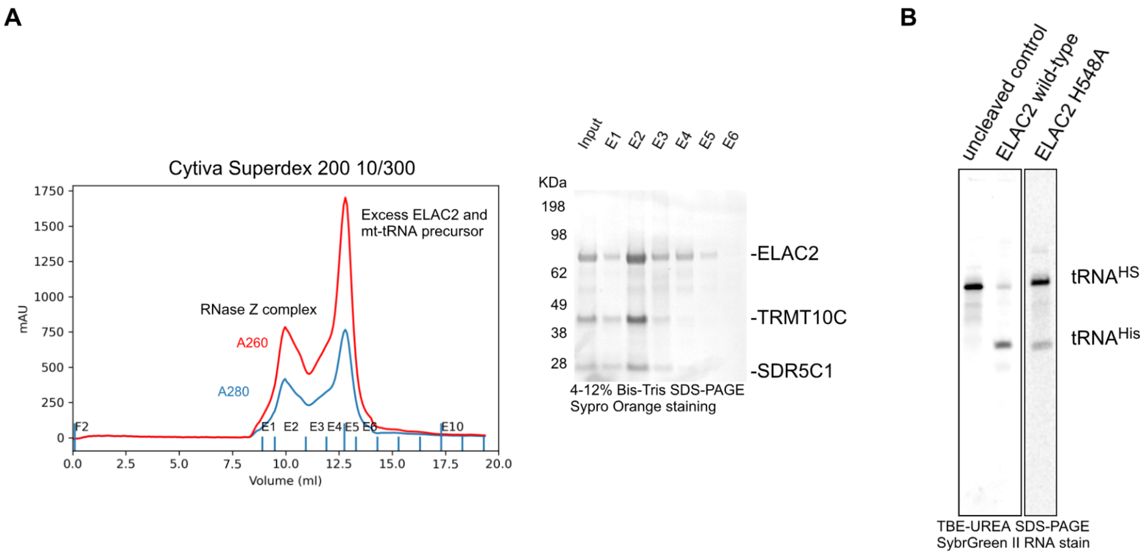

Appendix Figure S1 - Mitochondrial RNAse Z complex formation.

A TRMT10C/SDR5C1 was mixed with a twofold molar excess of ELAC2 and mt-tRNA-HS precursor, and run through size exclusion chromatography. The fractions E1-E6 were analyzed by SDS-PAGE stained for protein (right panel). Fraction E2 was used for cryo-EM grid preparation.

B TBE-UREA polyacrylamide gel electrophoresis of the samples used for grid preparation, with wild-type ELAC2 or the H548A mutant.

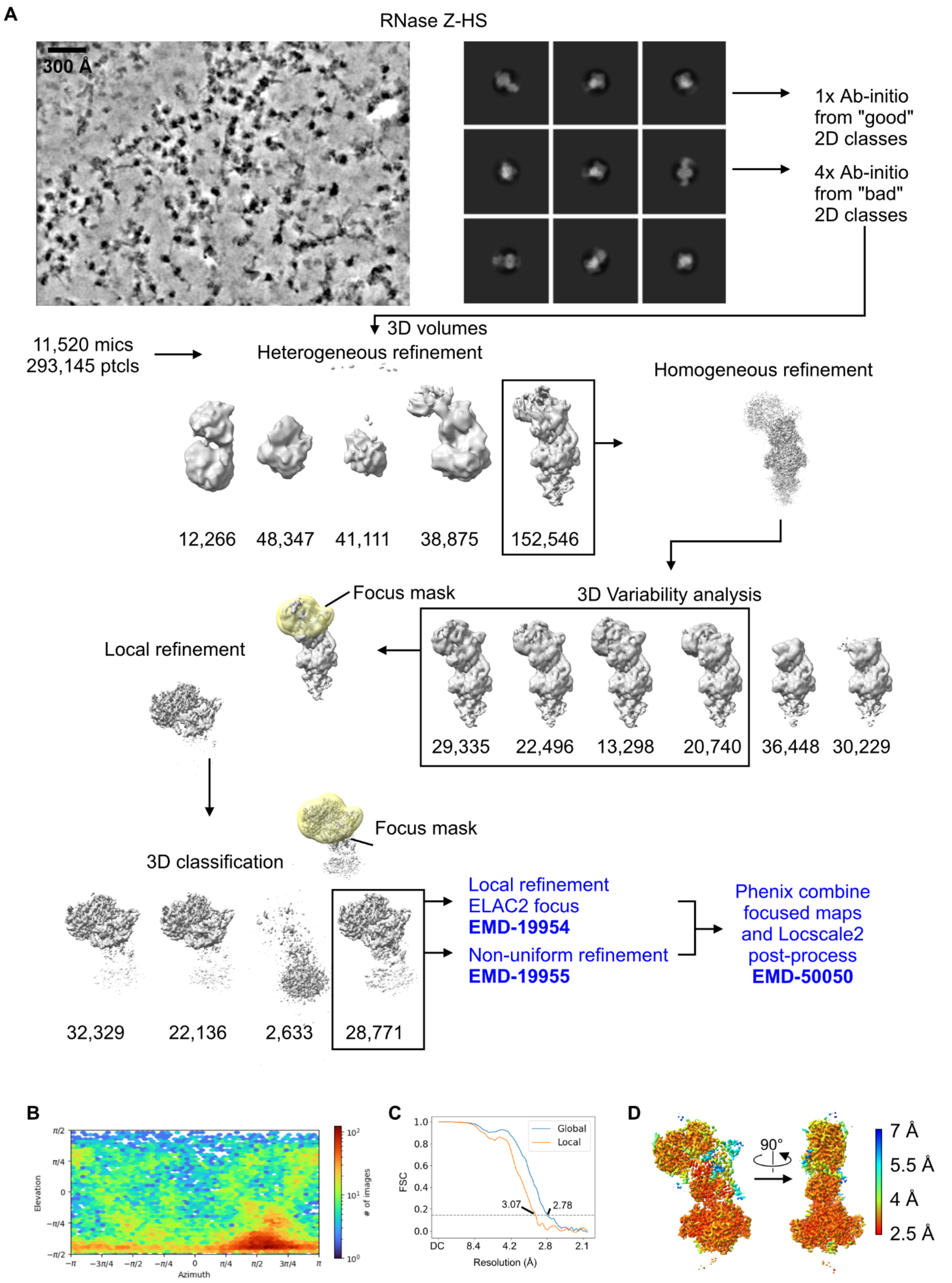
A
RNase Z-HS
300 Å
1x Ab-initio from "good" 2D classes
4x Ab-initio from "bad" 2D classes
3D volumes
11,520 mics
293,145 ptcls
Heterogeneous refinement
Homogeneous refinement
12,266
48,347
41,111
38,875
152,546
Focus mask
3D Variability analysis
Local refinement
29,335
22,496
13,298
20,740
36,448
30,229
Focus mask
3D classification
32,329
22,136
2,633
28,771
Local refinement
ELAC2 focus
EMD-19954
Non-uniform refinement
EMD-19955
Phenix combine focused maps and Locscale2 post-process
EMD-50050
B
Elevation
Azimuth
# of images
C
FSC
Global
Local
3.07
2.78
Resolution (Å)
DC
8.4
4.2
2.8
2.1
D
90°
7 Å
5.5 Å
4 Å
2.5 Å

Appendix Figure S2 - Cryo-EM data-processing for the RNase Z-HS dataset.

A Data processing strategy. All the micrographs were pre-processed in WARP. An example micrograph denoised in WARP is shown. The particles extracted by WARP were processed in CryoSPARC v4.1. Examples of good 2D classes are shown.

B Angular orientations of the particles used in the final reconstruction.

C Fourier Shell Correlation of the final global and local (ELAC2 focus) refinements.

D Composite map colored by local resolution, as calculated in CryoSPARC using the composite half maps. The masks for FSC calculation were generated in CryoSPARC using a relative threshold of 0.5.

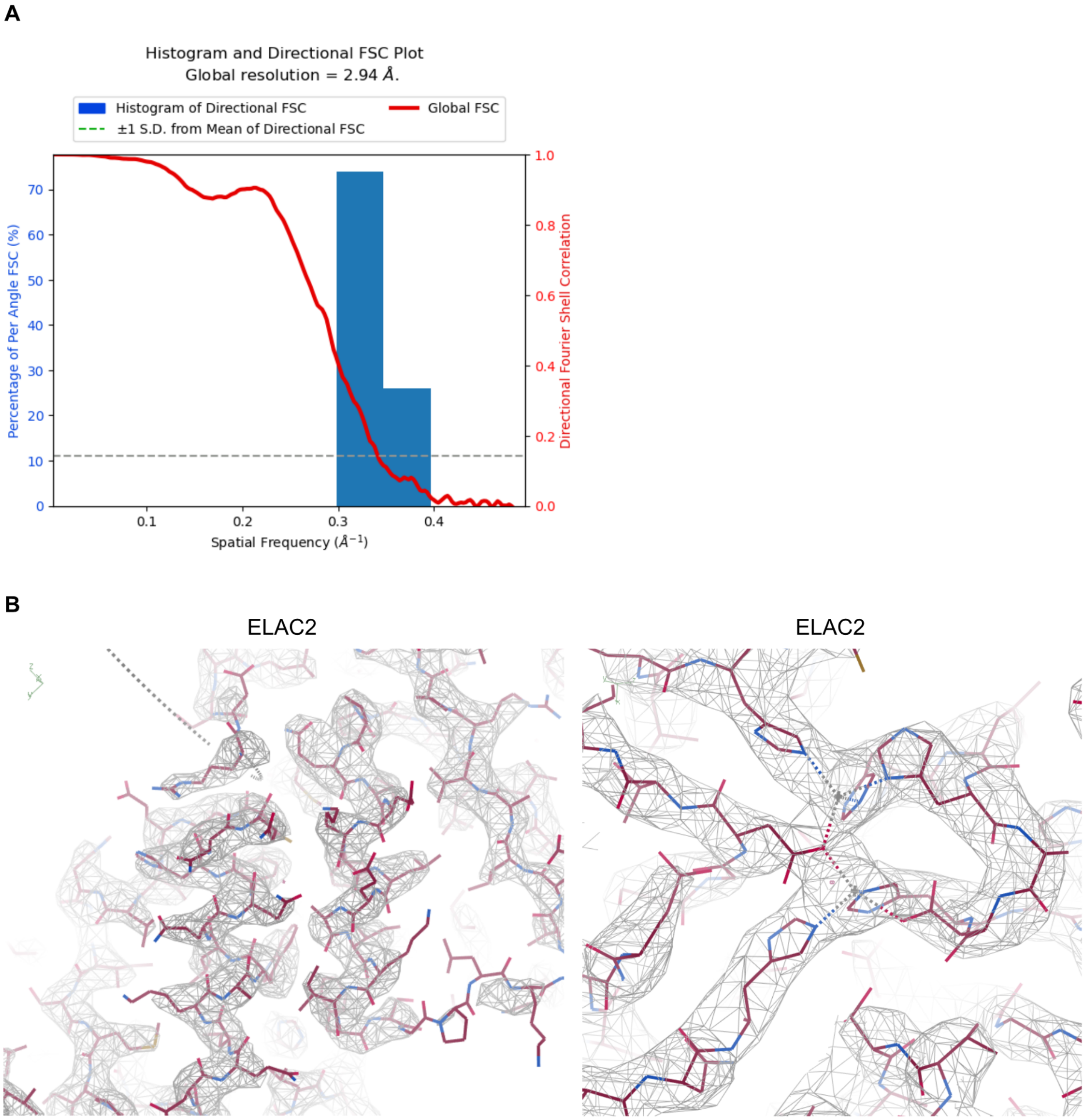

Appendix Figure S3 - Resolution of the RNase Z-HS cryo-EM structure (EMD-50050).

A Histograms and directional FSC plots for the EM density map calculated using 3DFSC (Tan et al., 2017). The composite half maps were provided as input (cone angle 20 degrees, FSC cutoff 0.143, Sphericity threshold 0.5, and high pass filter 150 Å). The mask for FSC calculation was generated in CryoSPARC using a relative threshold of 0.5.

B Representative densities (RMSD 2.0).

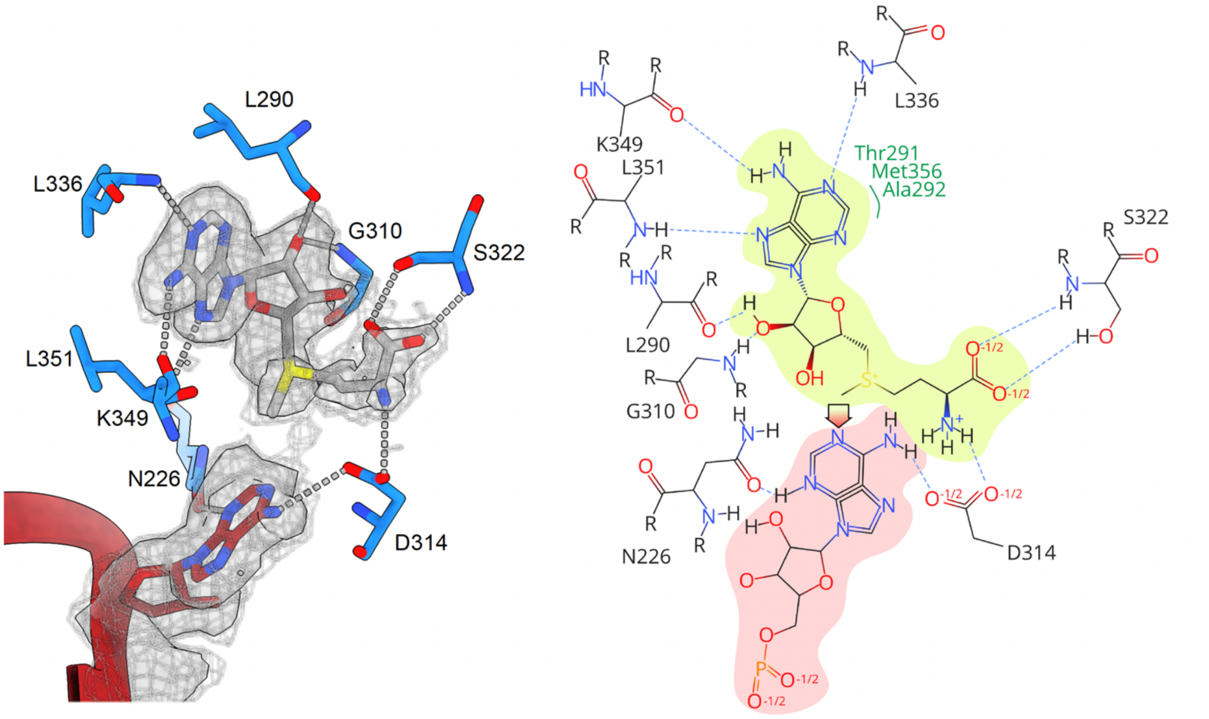

Appendix Figure S4 - TRMT10C active site close-up.

TRMT10C active site (blue), showing the cryo-EM density around SAM (gray) from a TRMT10C/SDR5C1 focused reconstruction (EMDB-51230; PDB 9GCH) and the position 9 adenine (red) as a gray transparent surface. The same density with a lower threshold (mesh) shows a peak for the A9 N1-methyl. H-bonds are indicated with gray dotted lines. The right panel shows the same interactions in a 2D diagram generated with PoseEdit (Diedrich et al, 2023) within ProteinsPlus (Schöning-Stierand et al, 2022). SAM and position 9 adenine have yellow and red outlines, respectively. The yellow-red arrow indicates the methyl group transfer from SAM.

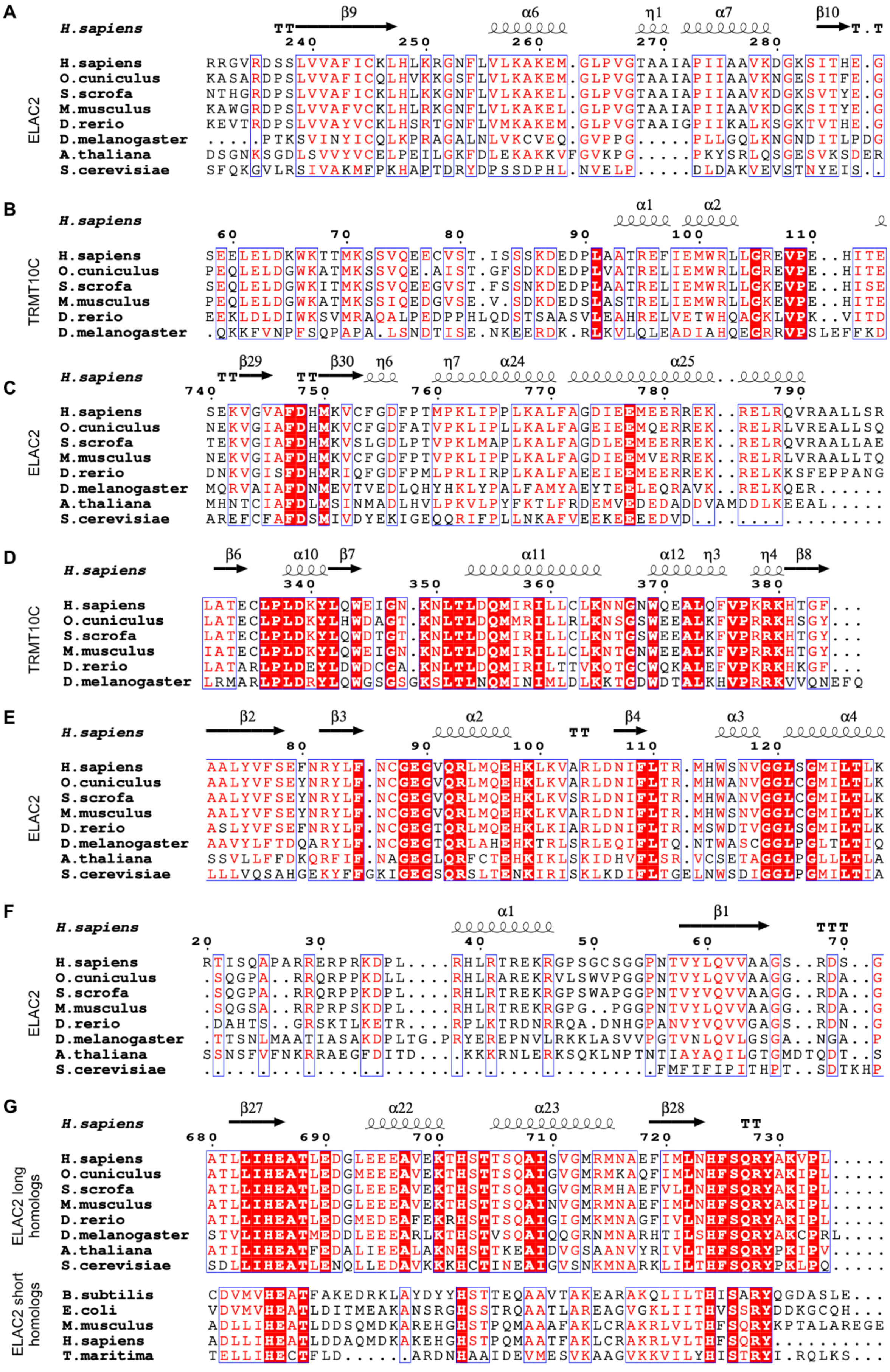

A
B
C
D
E
F
G
ELAC2
TRMT10C
ELAC2 long homologs
ELAC2 short homologs
H.sapiens
O.cuniculus
S.scrofa
M.musculus
D.rerio
D.melanogaster
A.thaliana
S.cerevisiae
B.subtilis
E.coli
T.maritima

Appendix Figure S5 - Structure-based multiple sequence alignment of ELAC2 and TRMT10C homologs.

A-G Multiple segments of the multiple sequence alignment. The alignments were generated with PROMALS3D (Pei et al., 2008). The UniProt IDs/RefSeq or NCBI and PDB codes (when available) are as follows. Long homologs: *H. sapiens* ELAC2 (Q9BQ52, RNase Z structure); *O. cuniculus* ELAC2 (NCBI: XM_002718909.4); *S. scrofa* ELAC2 (NCBI: NM_001243216.1); *M. musculus* ELAC2 (Q80Y81); *D. rerio* (NCBI: NP_001243133.1); *D. melanogaster* (Q8MKW7); *A. thaliana* ELAC2 (Q8VYS2); *S. cerevisiae* ELAC2 (P36159, PDB 5MTZ). Short homologs: *B. subtilis* RNase Z (P54548, PDB 1Y44); *E. coli* RNaseBN (P0A8V0); *M. musculus* ELAC1 (Q8VEB6); ELAC1 (Q9H777, PDB 3ZWF); *T. maritima* (Q9WZW8, PDB 2E7Y). TRMT10C homologs: *H. sapiens* (Q7L0Y3); *O. cuniculus* (G1T0M2); *S. scrofa* (F1SL15); *M. musculus* (Q3UFY8); *D. rerio* (B8JM40); *D. melanogaster* (Q7JUX9).

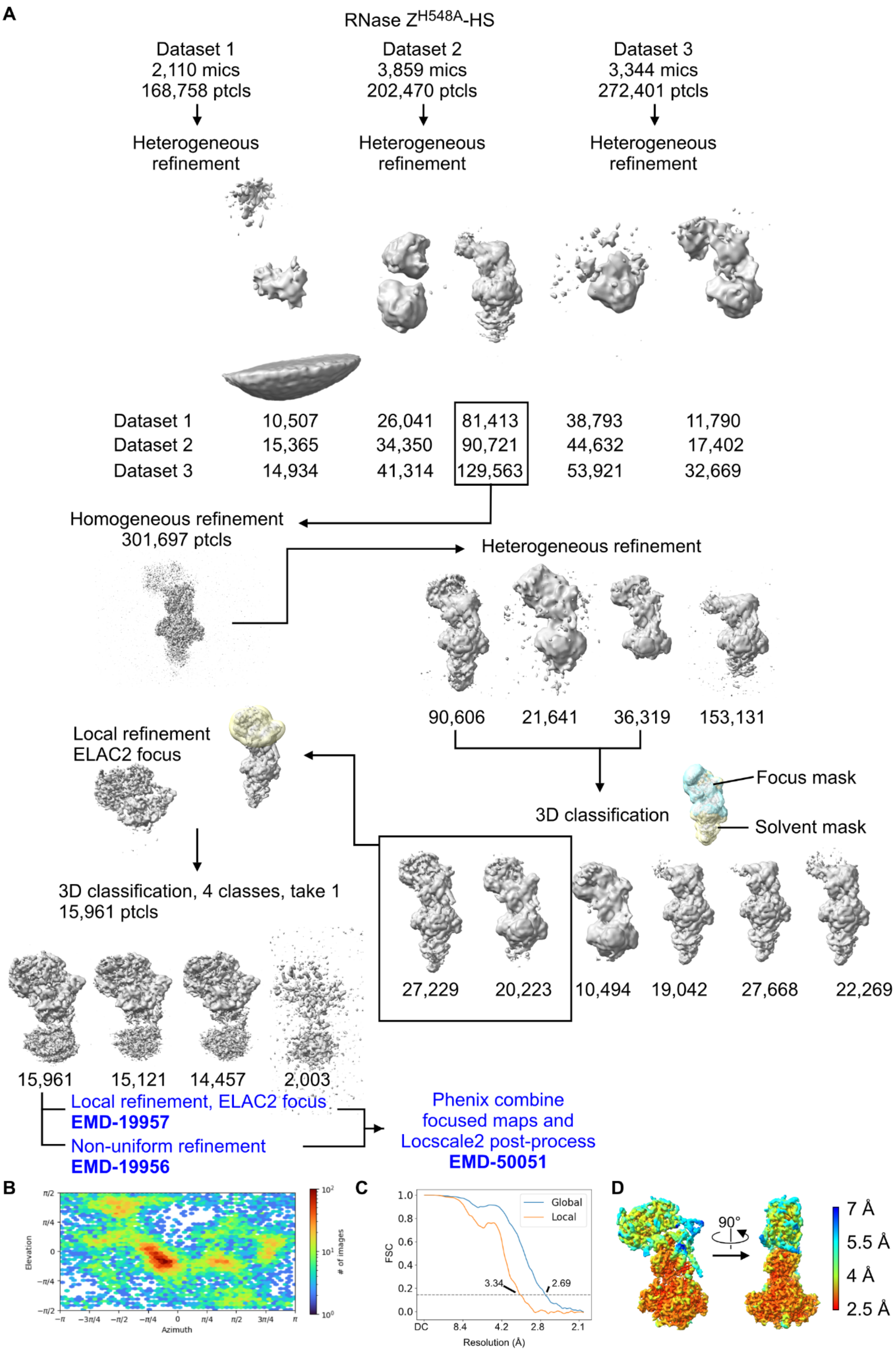

A
RNase Z^H548A-HS
Dataset 1
2,110 mics
168,758 ptcls
Dataset 2
3,859 mics
202,470 ptcls
Dataset 3
3,344 mics
272,401 ptcls
Heterogeneous refinement
Heterogeneous refinement
Heterogeneous refinement
Dataset 1 10,507 26,041 81,413 38,793 11,790
Dataset 2 15,365 34,350 90,721 44,632 17,402
Dataset 3 14,934 41,314 129,563 53,921 32,669
Homogeneous refinement
301,697 ptcls
Heterogeneous refinement
90,606 21,641 36,319 153,131
Local refinement
ELAC2 focus
3D classification
Focus mask
Solvent mask
27,229 20,223 10,494 19,042 27,668 22,269
3D classification, 4 classes, take 1
15,961 ptcls
15,961 15,121 14,457 2,003
Local refinement, ELAC2 focus
EMD-19957
Non-uniform refinement
EMD-19956
Phenix combine focused maps and Locscale2 post-process
EMD-50051
B
Elevation
Azimuth
# of images
C
FSC
Global
Local
3.34
2.69
Resolution (Å)
DC 8.4 4.2 2.8 2.1
D
90°
7 Å
5.5 Å
4 Å
2.5 Å

Appendix Figure S6 - Cryo-EM data-processing for the RNase Z^{H548A}-HS dataset.

A Data processing strategy. All the micrographs were pre-processed in WARP. Three datasets were pooled after independent heterogeneous refinement jobs, using the *ab-initio* volumes from RNase Z-HS (Appendix Fig S2) as 3D references.

B Angular orientations of the particles used in the final reconstruction.

C Fourier Shell Correlation of the final global and local (ELAC2 focus) refinements.

D Composite map colored by local resolution, as calculated in CryoSPARC using the composite half maps. The masks for FSC calculation were generated in CryoSPARC using a relative threshold of 0.5.

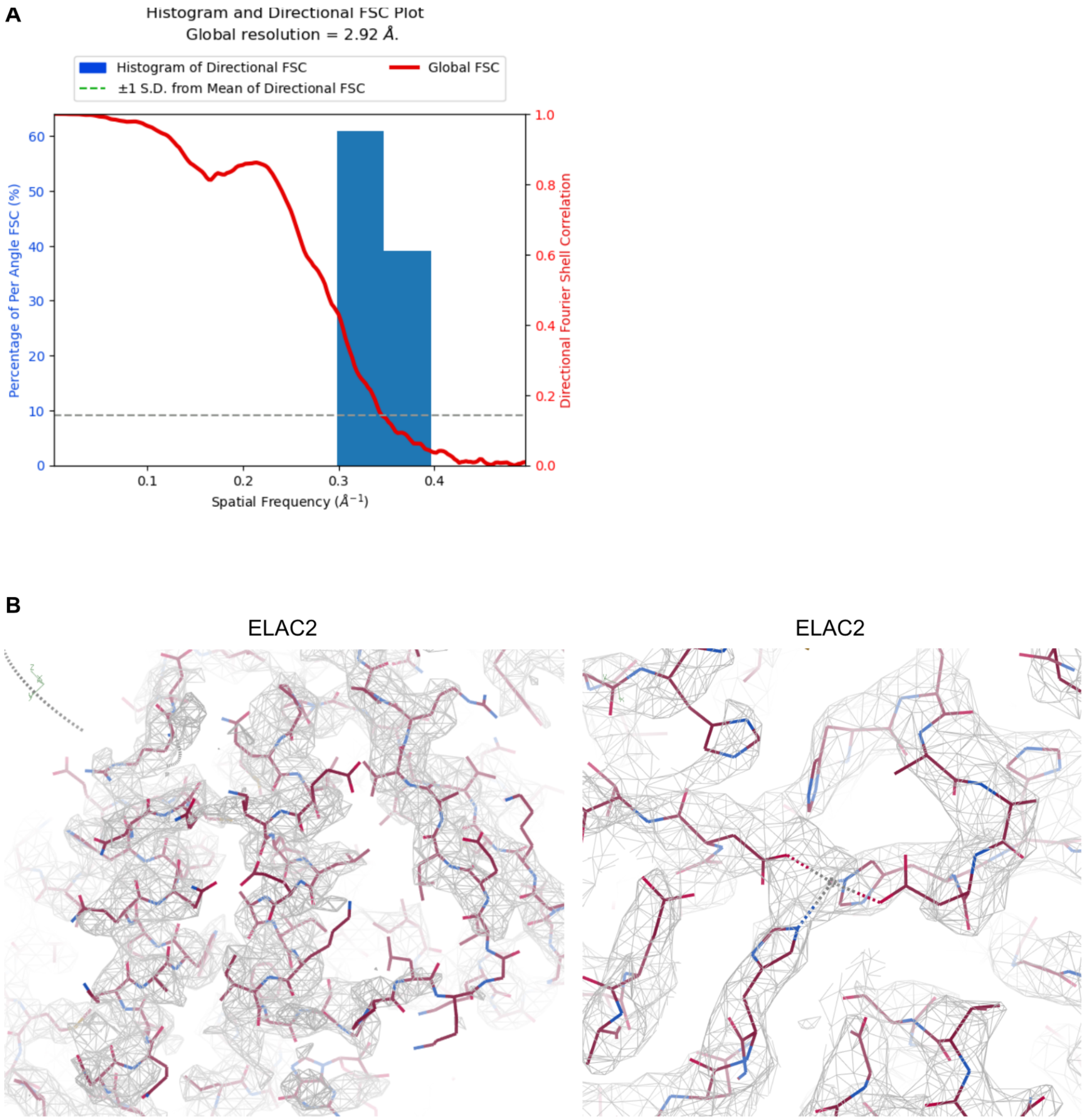

Appendix Figure S7 - Resolution of the RNase Z^{H548A}-HS cryo-EM structure (EMD-50051).

A Histograms and directional FSC plots for the EM density map were calculated using 3DFSC (Tan et al., 2017). The composite half maps were provided as input (cone angle 20 degrees, FSC cutoff 0.143, Sphericity threshold 0.5, and high pass filter 150 Å). The mask for FSC calculation was generated in CryoSPARC using a relative threshold of 0.5.

B Representative densities (RMSD 2.0).

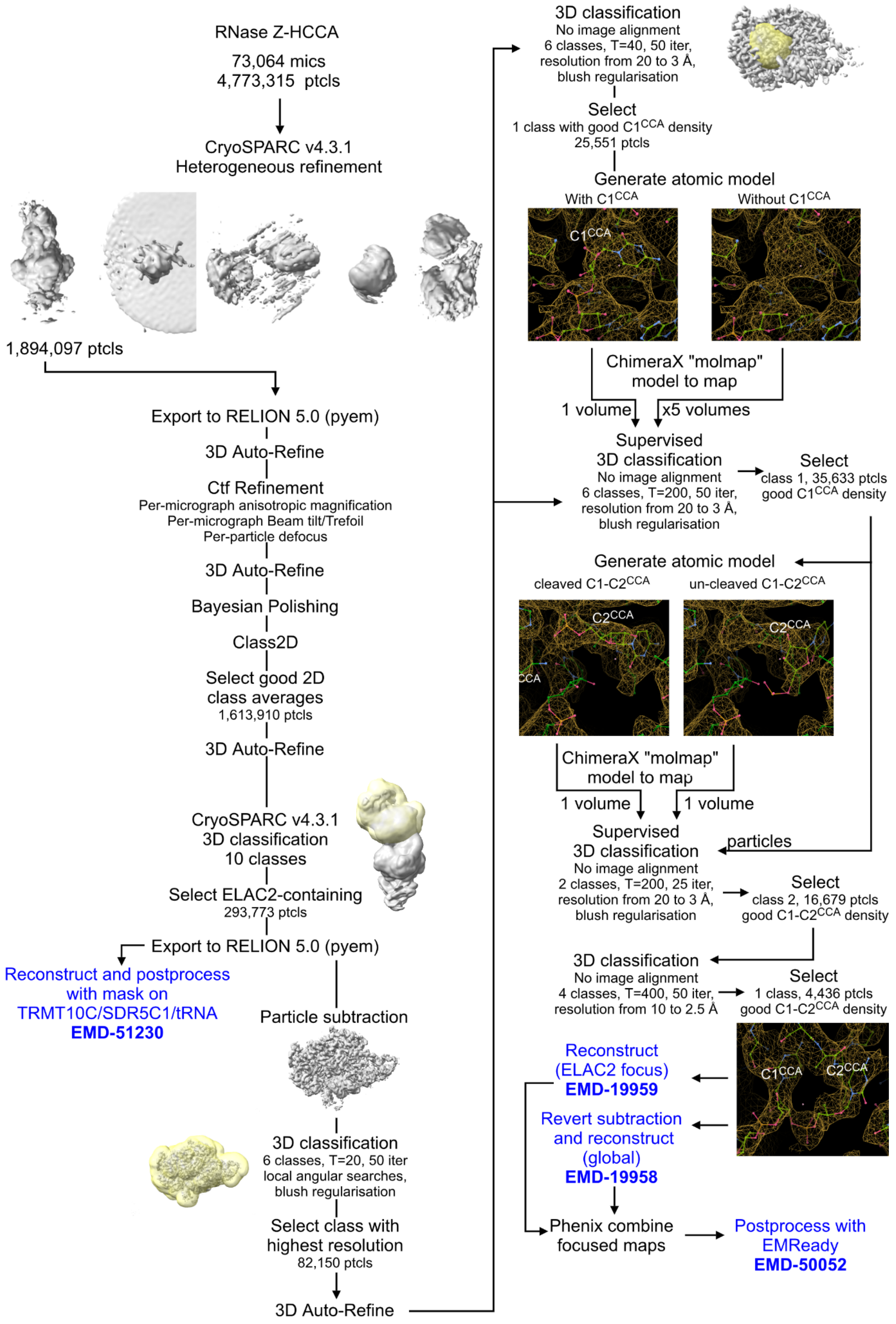
RNase Z-HCCA
73,064 mics
4,773,315 ptcls
CryoSPARC v4.3.1
Heterogeneous refinement
1,894,097 ptcls
Export to RELION 5.0 (pyem)
3D Auto-Refine
Ctf Refinement
Per-micrograph anisotropic magnification
Per-micrograph Beam tilt/Trefoil
Per-particle defocus
3D Auto-Refine
Bayesian Polishing
Class2D
Select good 2D
class averages
1,613,910 ptcls
3D Auto-Refine
CryoSPARC v4.3.1
3D classification
10 classes
Select ELAC2-containing
293,773 ptcls
Export to RELION 5.0 (pyem)
Reconstruct and postprocess
with mask on
TRMT10C/SDR5C1/tRNA
EMD-51230
Particle subtraction
3D classification
6 classes, T=20, 50 iter
local angular searches,
blush regularisation
Select class with
highest resolution
82,150 ptcls
3D Auto-Refine
3D classification
No image alignment
6 classes, T=40, 50 iter,
resolution from 20 to 3 Å,
blush regularisation
Select
1 class with good C1CCA density
25,551 ptcls
Generate atomic model
With C1CCA
Without C1CCA
C1CCA
ChimeraX "molmap"
model to map
1 volume
x5 volumes
Supervised
3D classification
No image alignment
6 classes, T=200, 50 iter,
resolution from 20 to 3 Å,
blush regularisation
Select
class 1, 35,633 ptcls
good C1CCA density
Generate atomic model
cleaved C1-C2CCA
un-cleaved C1-C2CCA
C2CCA
CCA
C2CCA
ChimeraX "molmap"
model to map
1 volume
1 volume
Supervised
3D classification
No image alignment
2 classes, T=200, 25 iter,
resolution from 20 to 3 Å,
blush regularisation
particles
Select
class 2, 16,679 ptcls
good C1-C2CCA density
3D classification
No image alignment
4 classes, T=400, 50 iter,
resolution from 10 to 2.5 Å
Select
1 class, 4,436 ptcls
good C1-C2CCA density
C1CCA
C2CCA
Reconstruct
(ELAC2 focus)
EMD-19959
Revert subtraction
and reconstruct
(global)
EMD-19958
Phenix combine
focused maps
Postprocess with
EMReady
EMD-50052

Appendix Figure S8 - Cryo-EM data-processing for the RNase Z-HCCA dataset.
All the micrographs were pre-processed in WARP. The particles extracted by WARP were processed in CryoSPARC v4.1 and further processed in RELION 5.0.

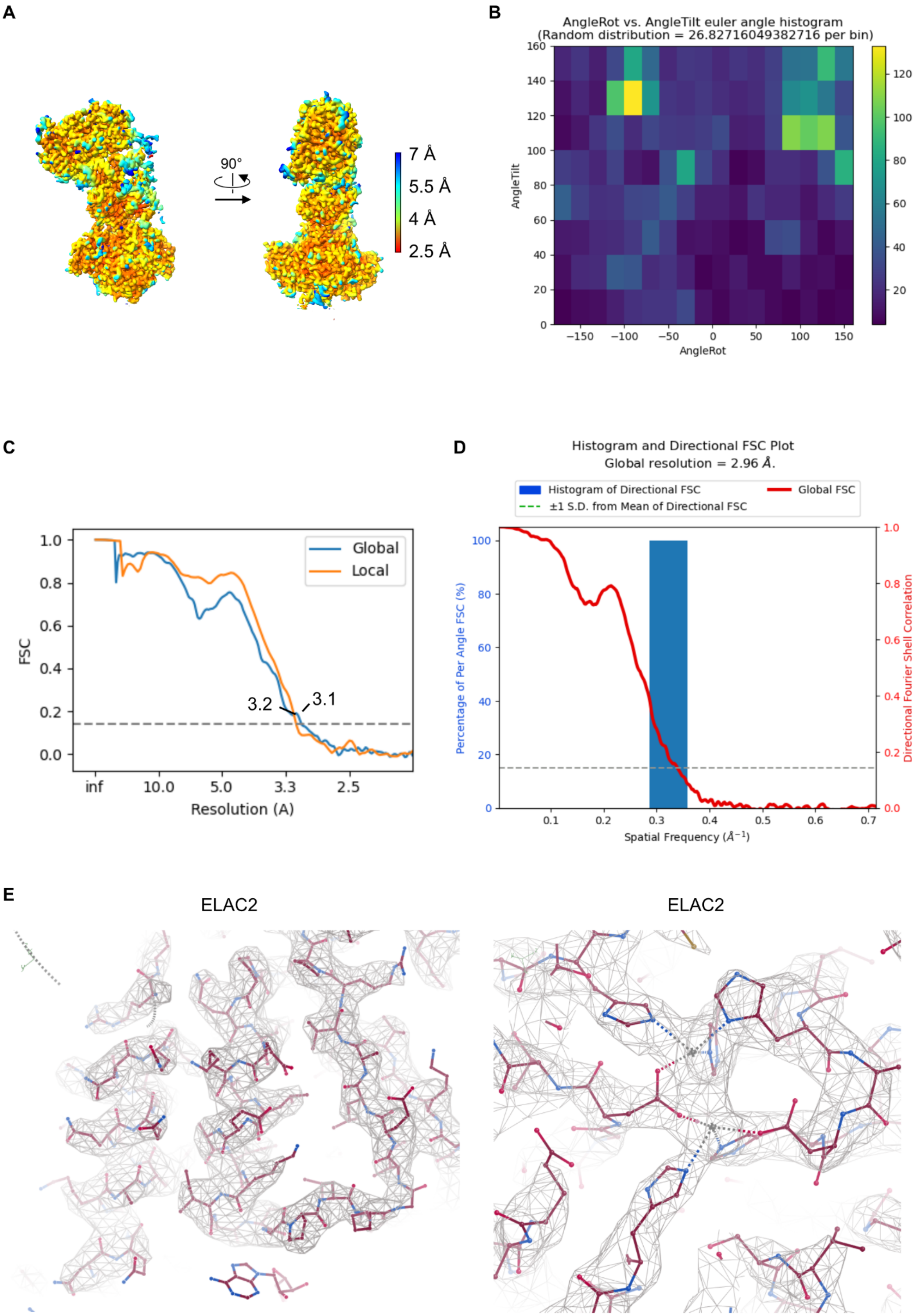
A
90°
7 Å
5.5 Å
4 Å
2.5 Å
B
AngleRot vs. AngleTilt euler angle histogram
(Random distribution = 26.82716049382716 per bin)
AngleTilt
AngleRot
C
FSC
Global
Local
3.2
3.1
Resolution (A)
inf
10.0
5.0
3.3
2.5
D
Histogram and Directional FSC Plot
Global resolution = 2.96 Å.
Histogram of Directional FSC
Global FSC
±1 S.D. from Mean of Directional FSC
Percentage of Per Angle FSC (%)
Directional Fourier Shell Correlation
Spatial Frequency (Å^{-1})
E
ELAC2
ELAC2

Appendix Figure S9 - Resolution of the RNase Z-HCCA cryo-EM structure (EMD-50052).

A Composite map colored by local resolution, as calculated in CryoSPARC using the composite half maps.

B Angular orientations of the particles used in the final reconstruction.

C Fourier Shell Correlation of the final global and local refinements calculated using RELION 5.0 post-process.

D Histograms and directional FSC plots for the EM density map were calculated using 3DFSC (Tan et al., 2017). The composite half maps were provided as input (cone angle 20 degrees, FSC cutoff 0.143, Sphericity threshold 0.5, and high pass filter 150 Å).

E Representative densities (RMSD 2.0). The masks for FSC calculation were generated in CryoSPARC using a relative threshold of 0.5.

A

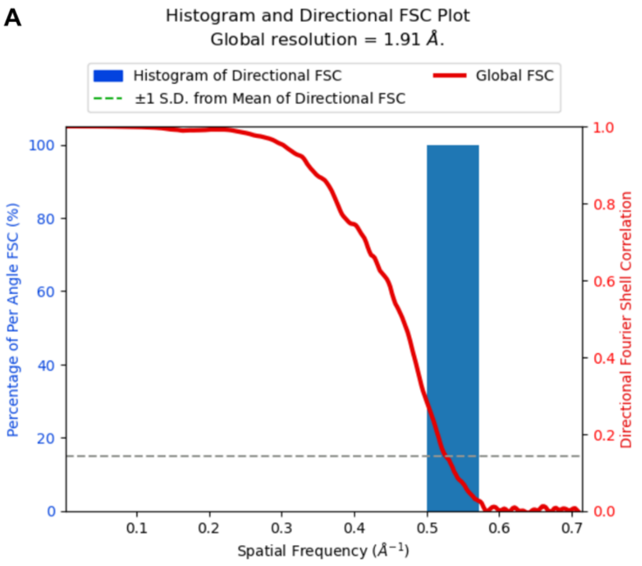

Histogram and Directional FSC Plot
Global resolution = 1.91 Å.
Histogram of Directional FSC
Global FSC
±1 S.D. from Mean of Directional FSC
Percentage of Per Angle FSC (%)
Directional Fourier Shell Correlation
Spatial Frequency (Å⁻¹)
0
20
40
60
80
100
0.0
0.2
0.4
0.6
0.8
1.0
0.1
0.2
0.3
0.4
0.5
0.6
0.7

B

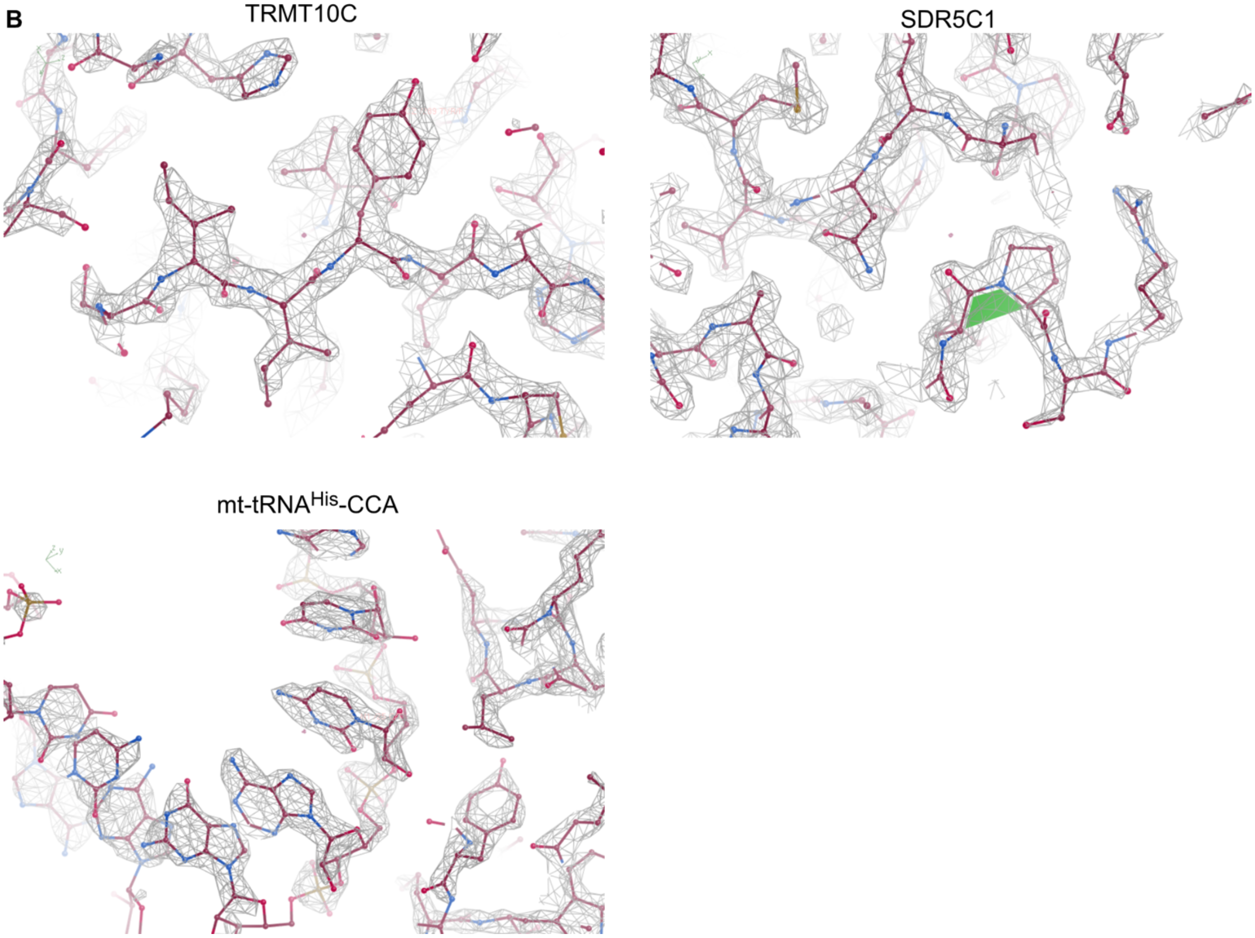

TRMT10C
SDR5C1
mt-tRNA^{His}-CCA

Appendix Figure S10 - Resolution of the RNase Z-HCCA cryo-EM structure (TRMT10C/SDR5C1 focus, EMD-51230).

A Histograms and directional FSC plots for the EM density map calculated using 3DFSC (Tan et al., 2017) (cone angle 20 degrees, FSC cutoff 0.143, Sphericity threshold 0.5, and high pass filter 150 Å). The mask for FSC calculation was generated in CryoSPARC using a relative threshold of 0.5.
B Representative densities (RMSD 3.0).
