# Supplementary figures and images for: Structural basis of 3′-tRNA maturation by the human mitochondrial RNase Z complex
Source: EMBO J. 2024 Nov 8;43(24):15. doi: 10.1038/s44318-024-00297-w (PMC11649783; doi:10.1038/s44318-024-00297-w)

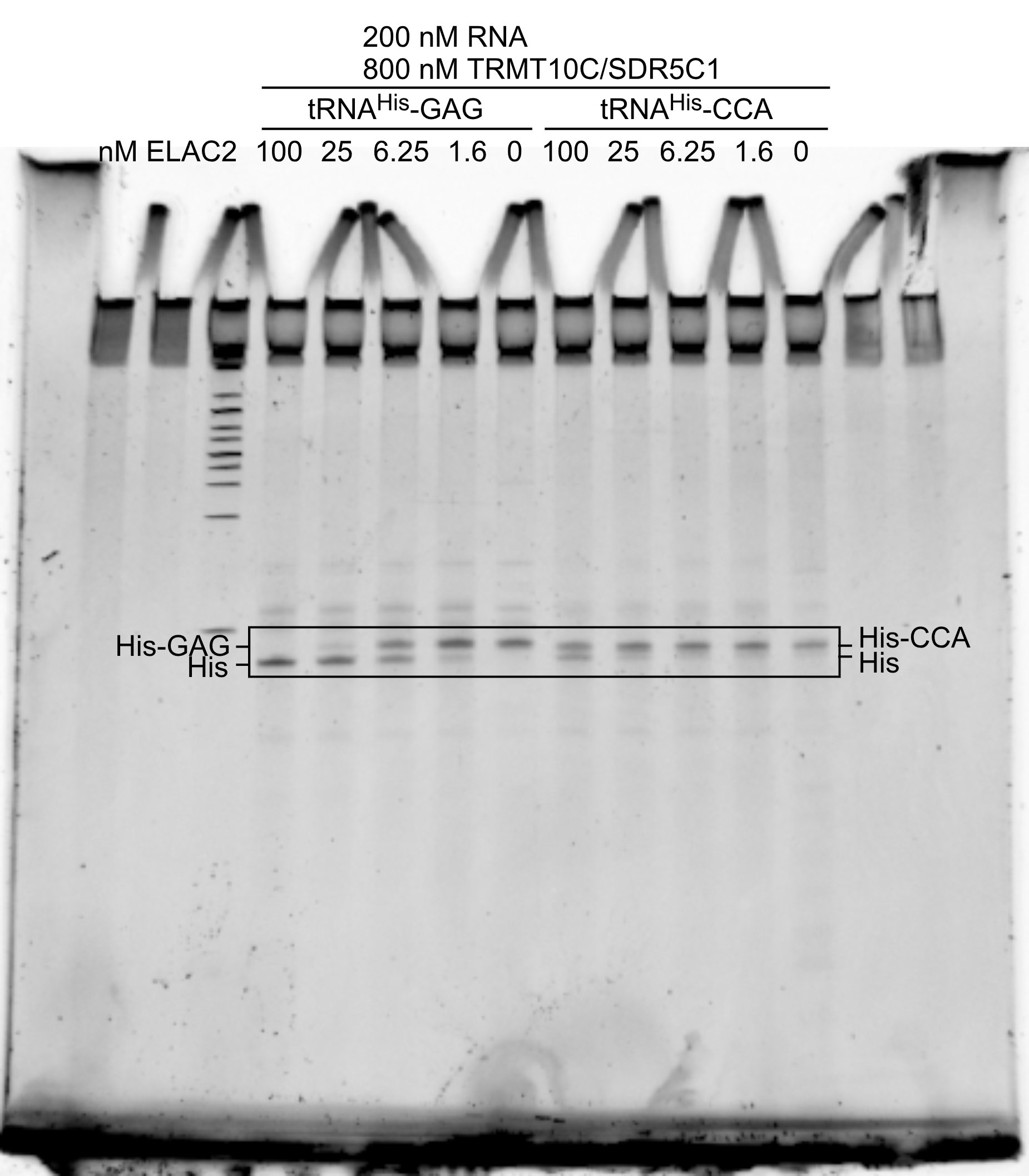

Supplement: Supplementary file 3 — Source data Fig. 5 [file 44318_2024_297_MOESM3_ESM.zip › 5A/Figure5A_uncropped.png]

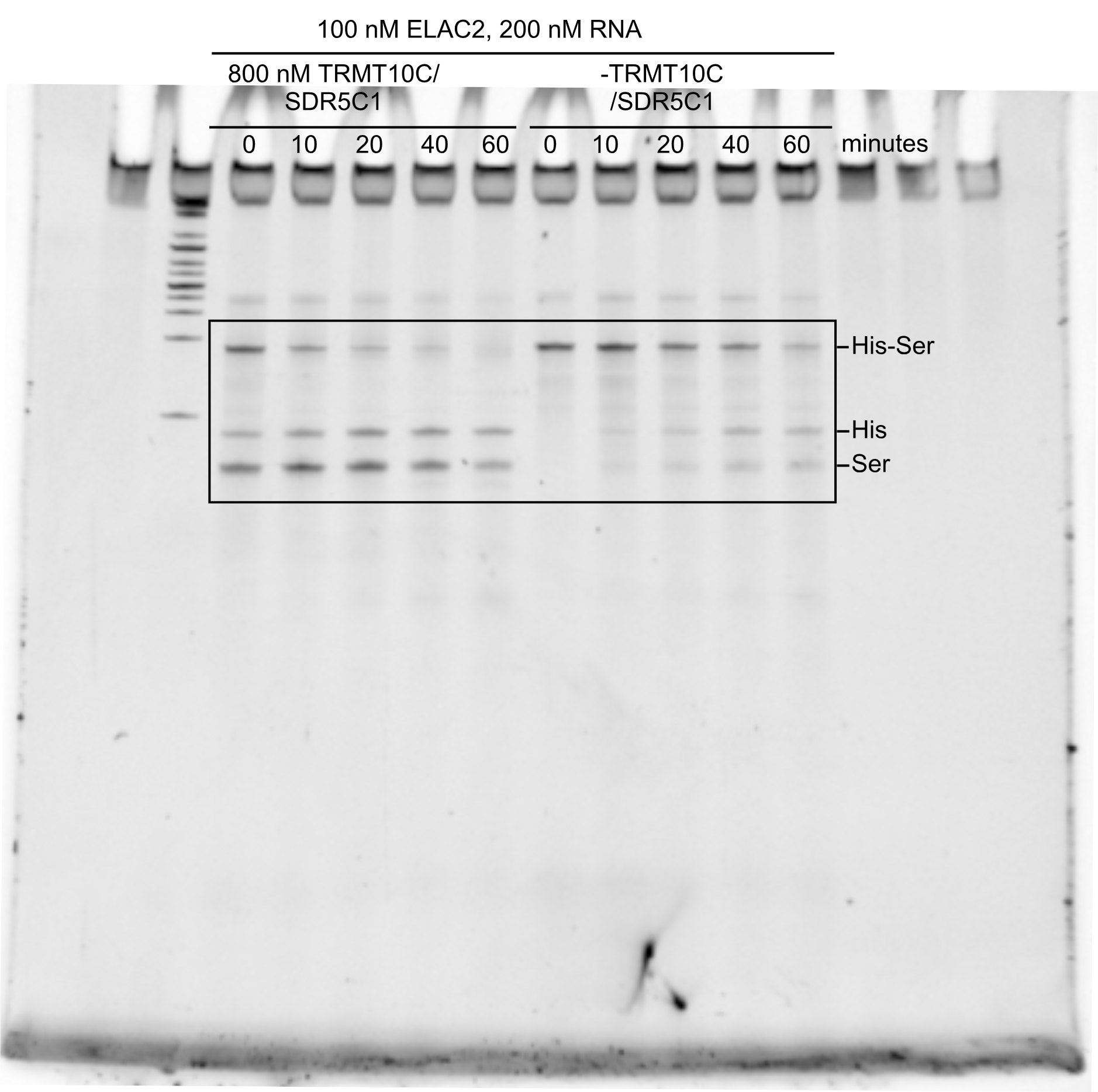

Supplement: Supplementary file 4 — EV Figure Source Data [file 44318_2024_297_MOESM4_ESM.zip › EV-Figure-Source-Data/FigureEV1A_uncropped.png]

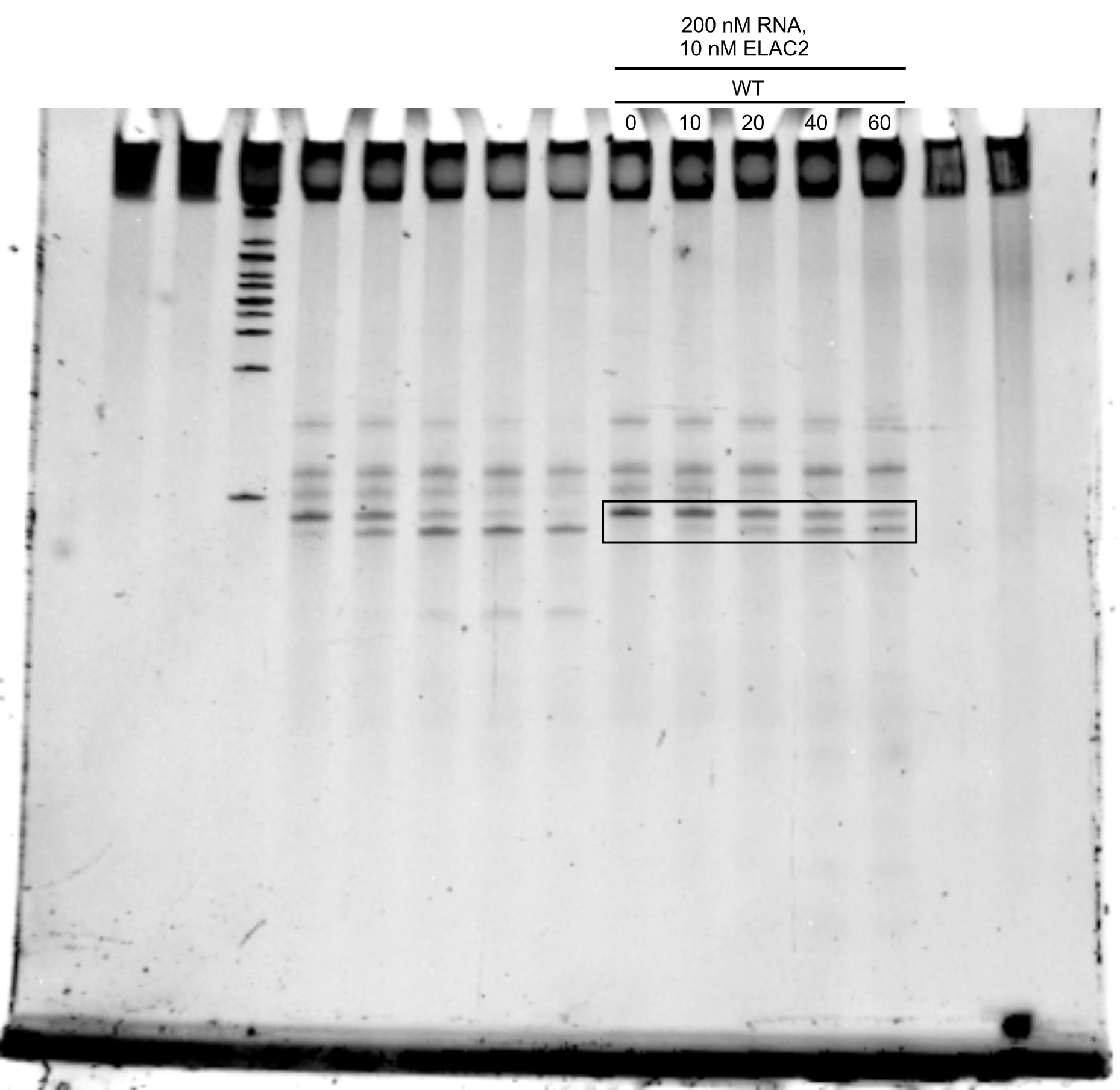

Supplement: Supplementary file 4 — EV Figure Source Data [file 44318_2024_297_MOESM4_ESM.zip › EV-Figure-Source-Data/FigureEV2A_1_uncropped.png]

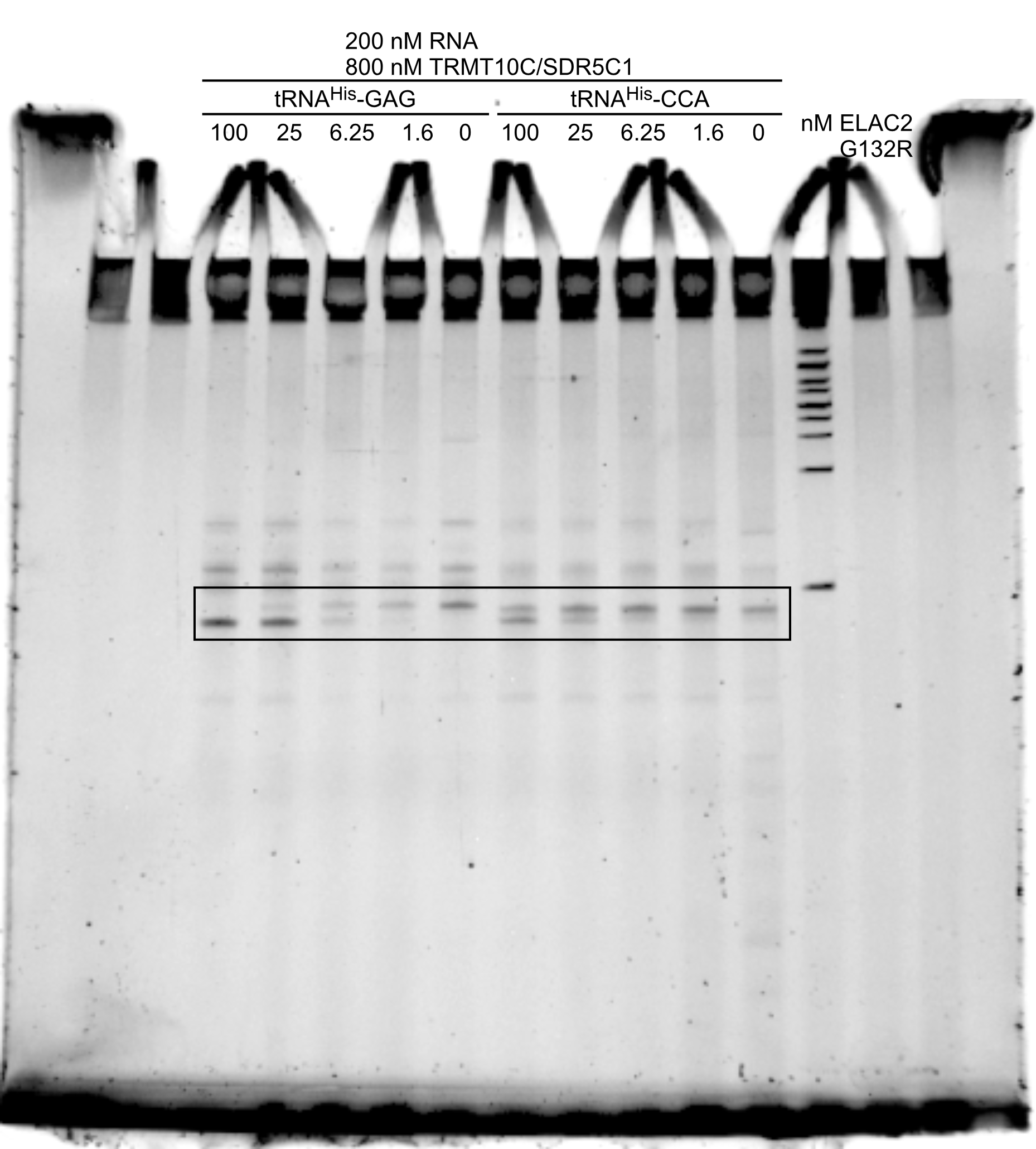

Supplement: Supplementary file 4 — EV Figure Source Data [file 44318_2024_297_MOESM4_ESM.zip › EV-Figure-Source-Data/FigureEV4_A_G132R.png]

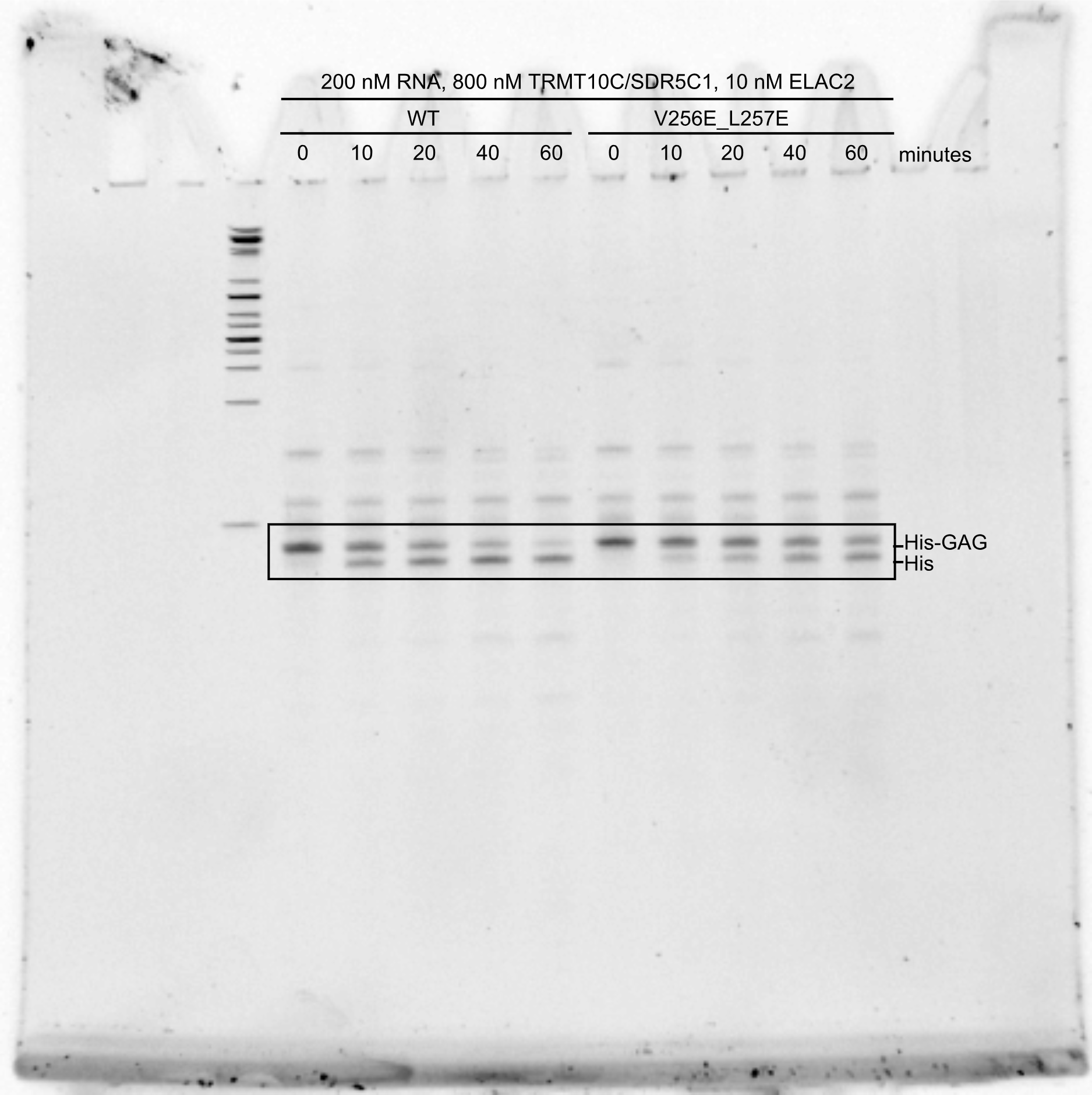

Supplement: Supplementary file 4 — EV Figure Source Data [file 44318_2024_297_MOESM4_ESM.zip › EV-Figure-Source-Data/FigureEV2A_2_uncropped.png]

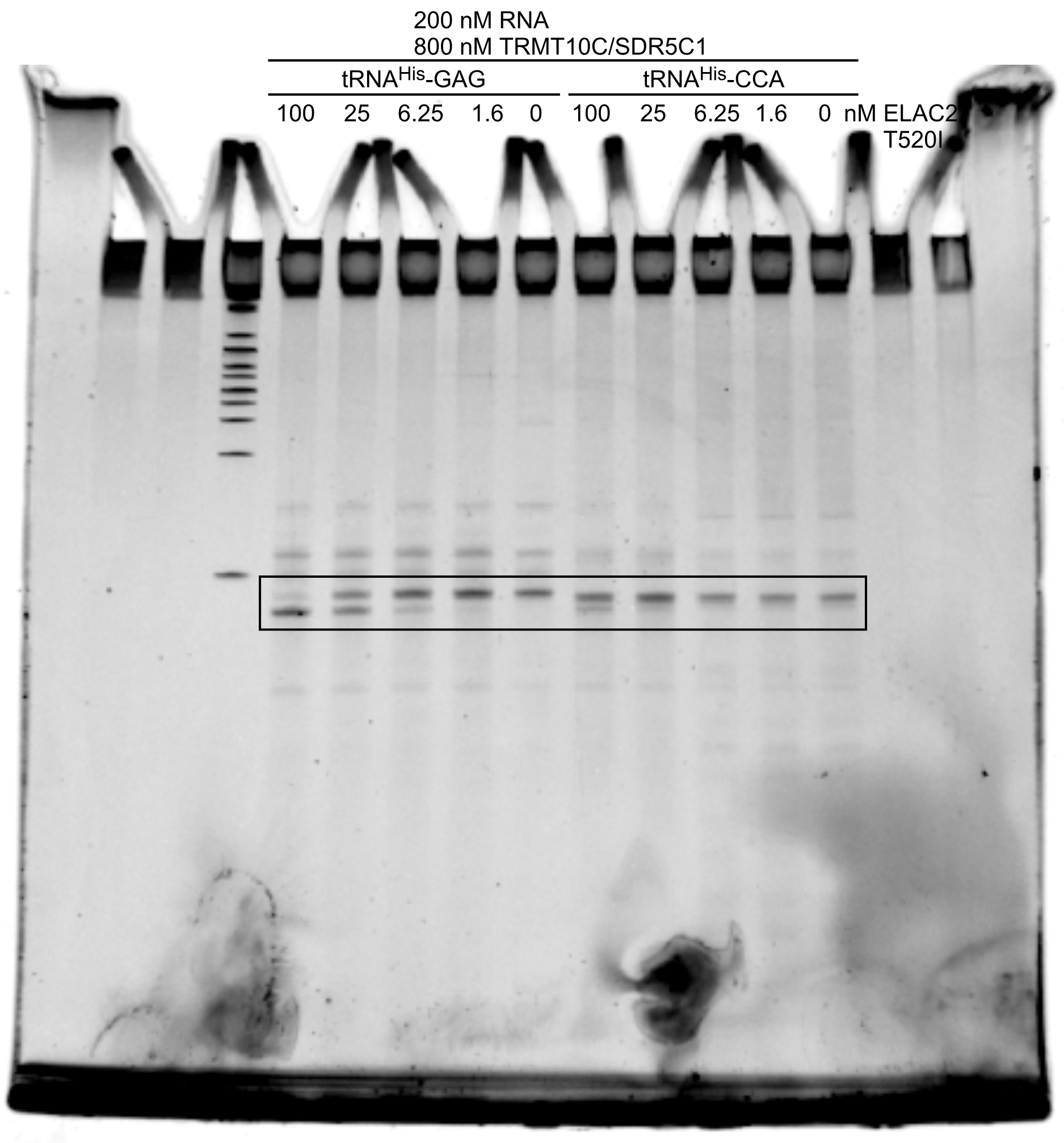

Supplement: Supplementary file 4 — EV Figure Source Data [file 44318_2024_297_MOESM4_ESM.zip › EV-Figure-Source-Data/FigureEV4_A_T520I.png]

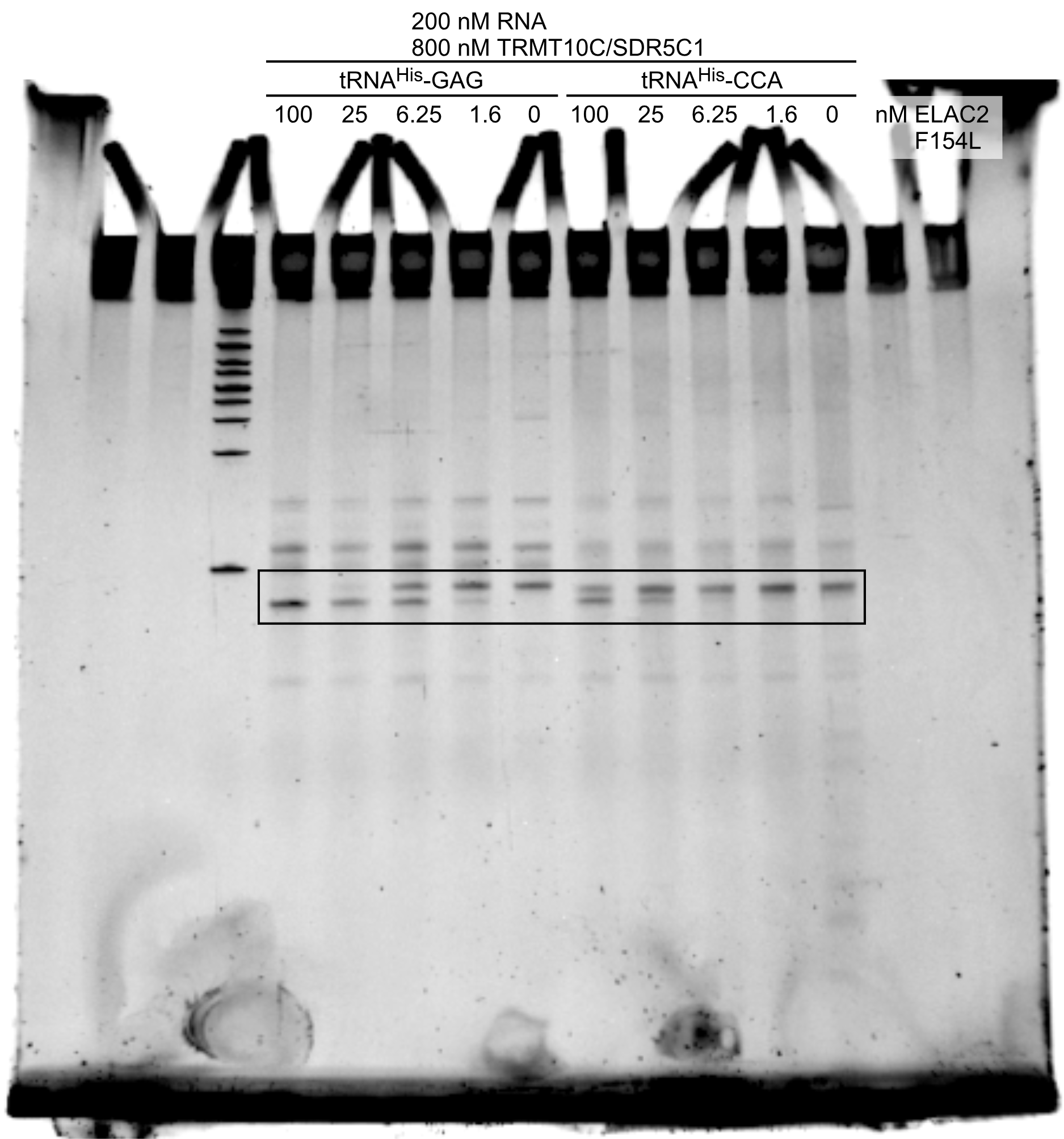

Supplement: Supplementary file 4 — EV Figure Source Data [file 44318_2024_297_MOESM4_ESM.zip › EV-Figure-Source-Data/FigureEV4_A_F154L.png]
