# Supplementary material for: Structural basis of 3′-tRNA maturation by the human mitochondrial RNase Z complex
Source: EMBO J. 2024 Nov 8;43(24):15. doi: 10.1038/s44318-024-00297-w (PMC11649783; doi:10.1038/s44318-024-00297-w)
Supplement: Supplementary file 5 — Expanded View Figures [file 44318_2024_297_MOESM5_ESM.pdf]

Expanded View Figures

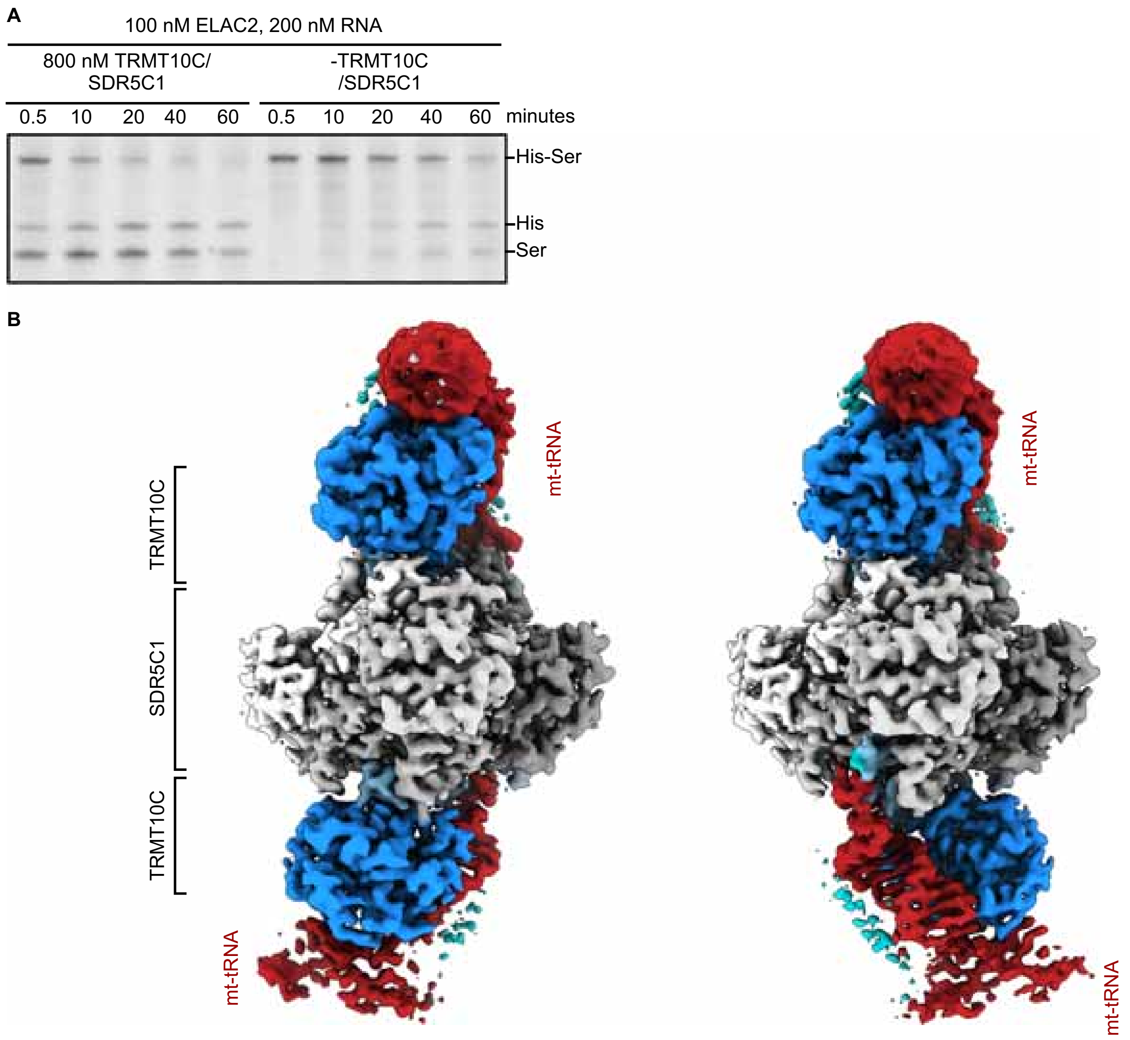

Figure EV1. The TRMT10C structure (related to Fig. 1).

(**A**) ELAC2 activity in the presence and absence of the TRMT10C/SDR5C1 platform. (**B**) Cryo-EM density of particles containing two TRMT10C subunits. The 4xSDR5C1 platform offers two mt-tRNA/TRMT10C binding sites. The platform has a vertical and a horizontal twofold symmetry axis. Consequently, it supports two mt-tRNA/TRMT10C subcomplexes, which can be in two different but equivalent orientations (left and right panels). The 4xSDR5C1 are colored in different shades of gray; TRMT10C is colored in dark blue with the NTD highlighted in light blue; the mt-tRNA is in red. ELAC2 density is not visible due to the low occupancy. Source data are available online for this figure.

 © The Author(s)

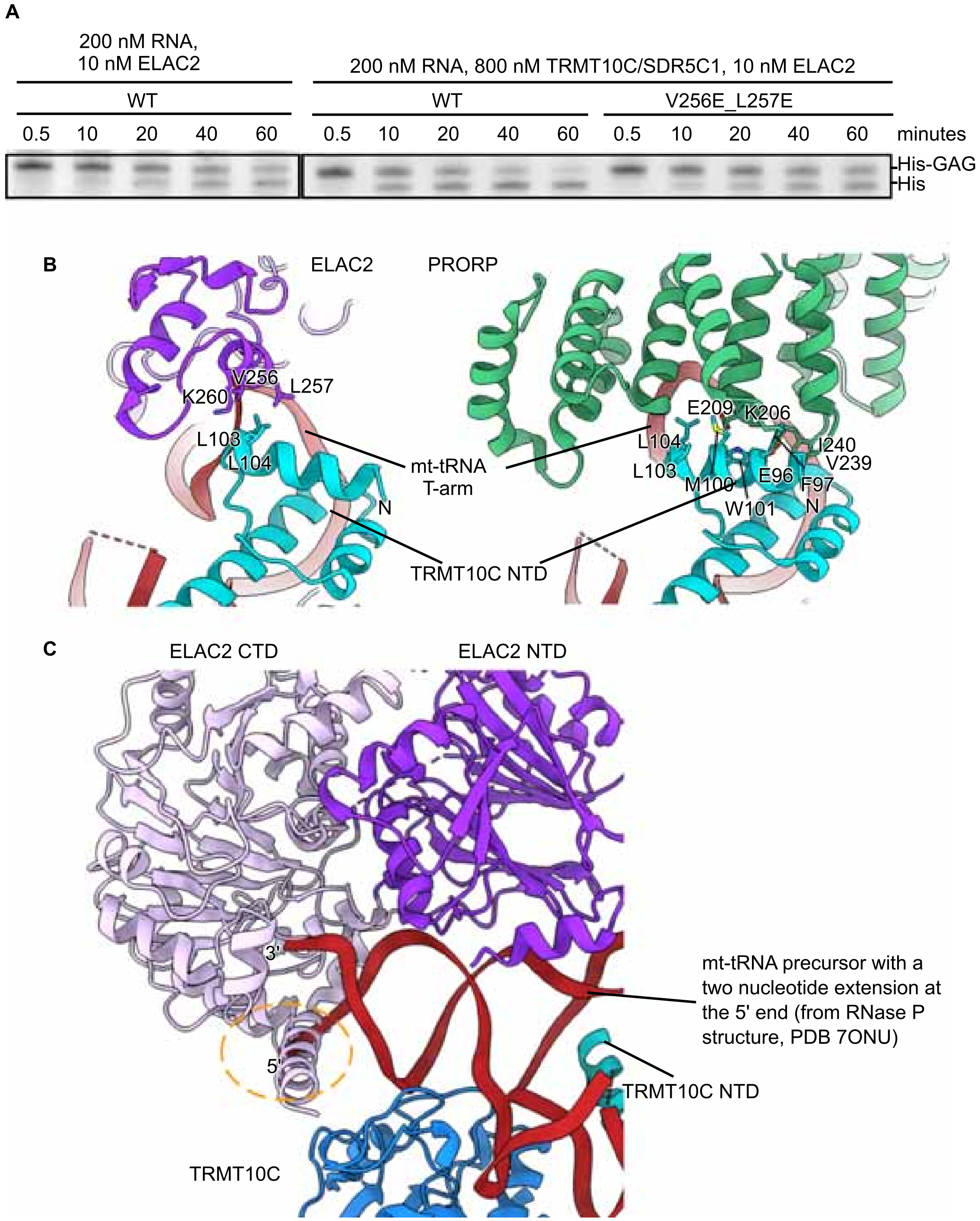

Figure EV2. TRMT10C/SDR5C1 interactions with ELAC2 and PRORP (related to Fig. 2).

(**A**) ELAC2 cleavage of the mt-tRNA^{His}-GAG precursor in the absence of TRMT10C/SDR5C1, or with the mutations V256E_L257E that affect residues interacting with TRMT10C. (**B**) TRMT10C NTD interacts with ELAC2 (RNase Z, left panel) and PRORP (RNase P, right panel) in the T loop region. (**C**) The ELAC2 C-terminal helix would collide with a 5′ extension on the mt-tRNA precursor (yellow circle). To make this figure, the mt-tRNA precursor in RNase Z was replaced with the RNase P one (mt-tRNA^{Tyr}), which has a 2-nucleotide 5′-extension. For this, The RNase Z structure was superimposed on the RNase P structure (Bhatta et al, 2021; PDB 7ONU), using TRMT10C as a reference. Source data are available online for this figure.

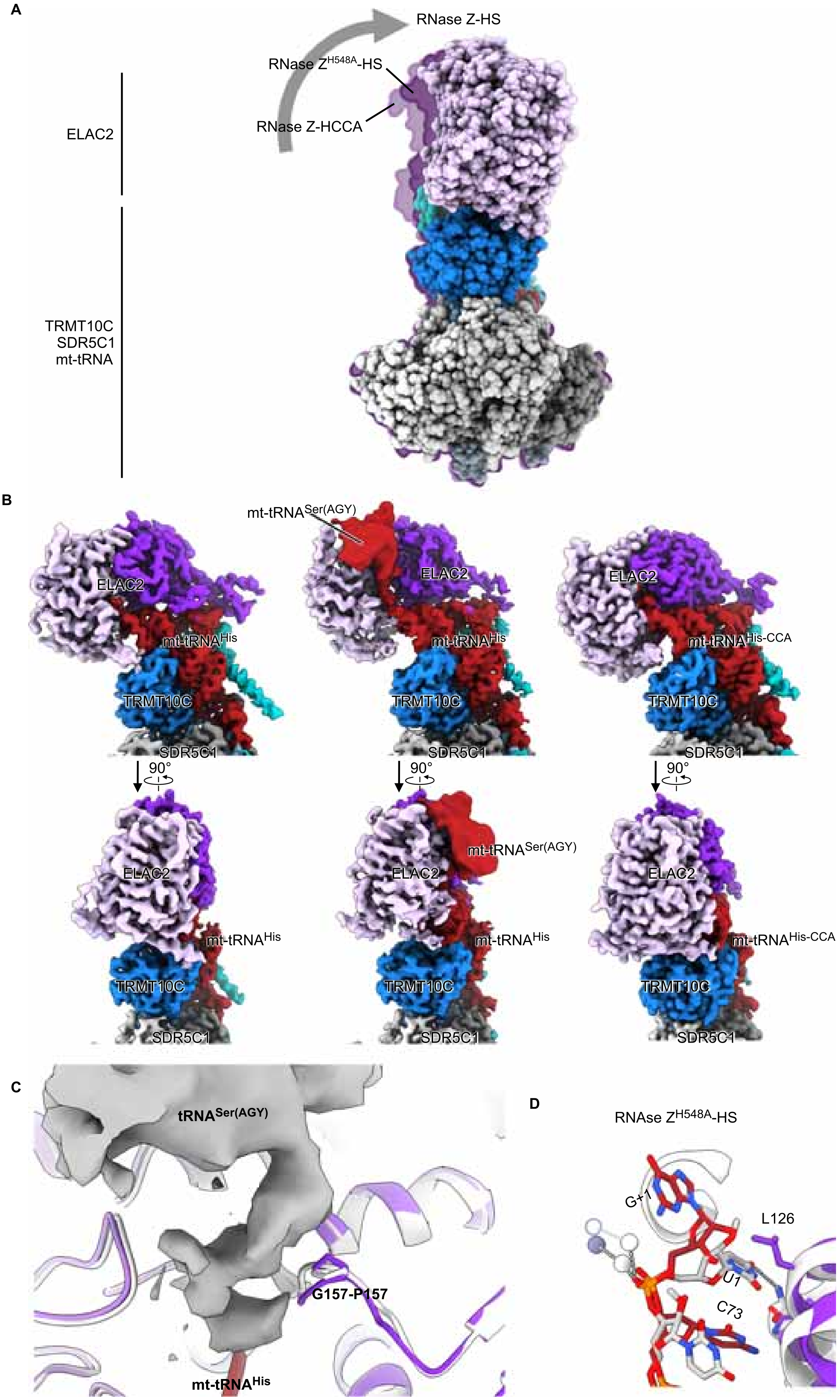
A
RNase Z-HS
RNase Z^H548A-HS
RNase Z-HCCA
ELAC2
TRMT10C
SDR5C1
mt-tRNA
B
mt-tRNA^Ser(AGY)
ELAC2
mt-tRNA^His
mt-tRNA^His-CCA
TRMT10C
SDR5C1
90°
C
tRNA^Ser(AGY)
G157-P157
mt-tRNA^His
D
RNAse Z^H548A-HS
G+1
L126
U1
C73

Figure EV3. Pivoting of ELAC2 on the mt-tRNA (related to Fig. 3).

(**A**) ELAC2 pivots on the mt-tRNA to reach the catalytic position. The RNase Z-HS structure is at the forefront. The outline of the RNase Z^{H548A}-HS and the RNase Z-HCCA structures are shown to highlight the movement of ELAC2. ELAC2 is colored in light and dark purple for the CTD and NTD, respectively, while TRMT10C is in blue, and the four SDR5C1 subunits are in shades of gray. (**B**) Cryo-EM composite maps of RNase Z bound to the three different tRNA precursors, aligned on TRMT10C. The cryo-EM density of the mt-$tRNA^{Ser(AGY)}$ moiety has been Gaussian-filtered in UCSF ChimeraX with a B-factor of 600 for better visualization. To visualize the ELAC2 movement, notice the changes in the gap with TRTM10C. (**C**) Cryo-EM density for the mt-$tRNA^{Ser(AGY)}$ in RNase Z^{H548A}-HS structure. The density, for which it was not possible to create an atomic model, displaces the G157-P157 loop compared with the RNase Z-HS structure (superposed in white). (**D**) Comparison with the *B. subtilis* RNase Z bound to precursor tRNA. In *B. Subtilis* (white), U1 inserts into a pocket and helps position the scissile bond. In RNase Z^{H548A}-HS, G1 does not bind in the pocket as it is partially occluded by L126. The *B. Subtilis* Zn^{2+} are shown as white spheres. The RNase Z^{H548A}-HS missing Zn^{2+} is drawn based on the RNase Z-HS structure.

© The Author(s)

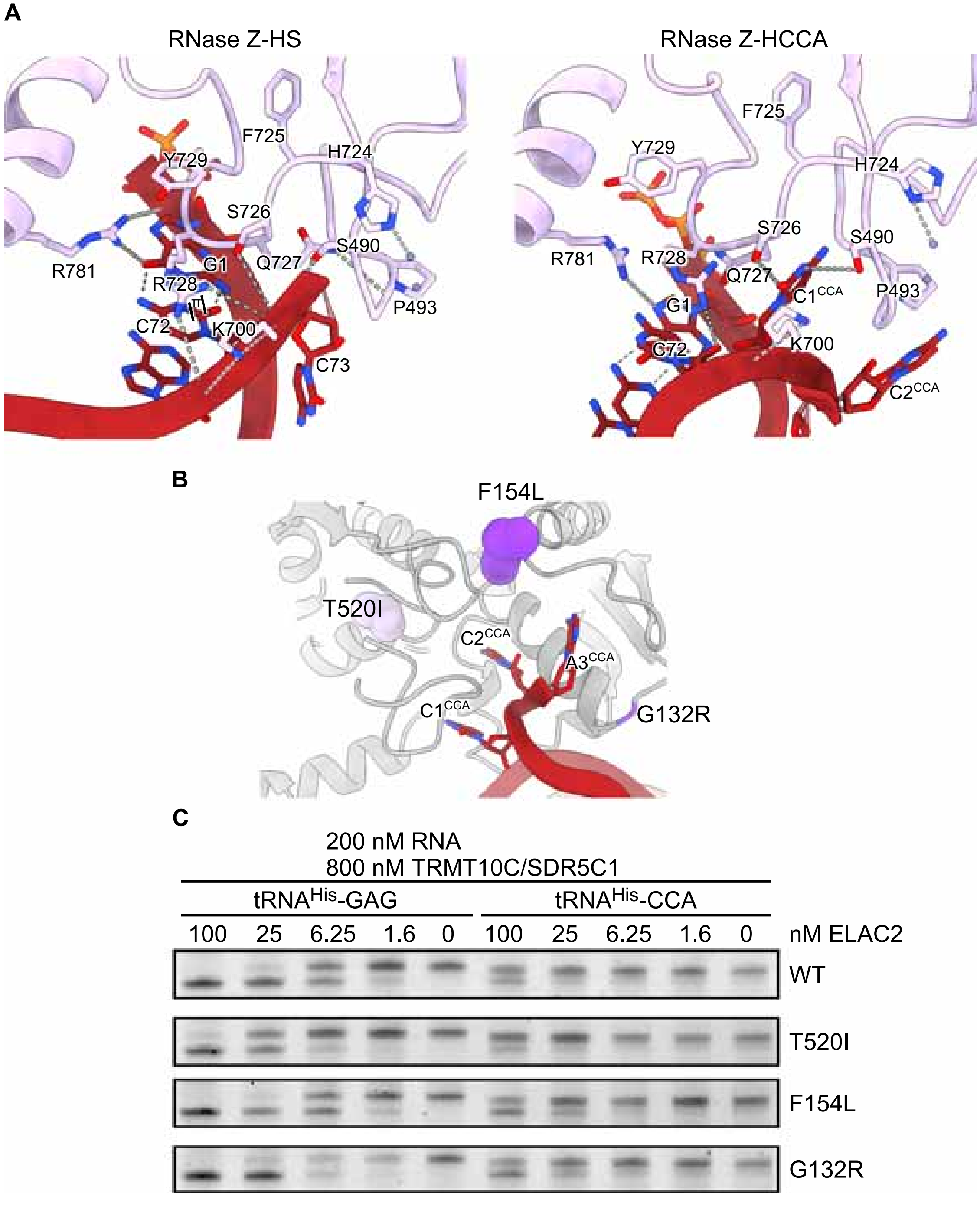

Figure EV4. Selected ELAC2 mutations involved in hypertrophic cardiomyopathy do not impair the 3′-CCA antideterminantion (related to Fig. 5).

(**A**) Interactions around the tRNA 3′-end or the 3′-CCA tail involve the same ELAC2 residues. Two parallel black lines indicate π-stacking. Selected electrostatic interactions are indicated with gray dotted lines. (**B**) Selected clinical mutations near the ELAC2 active site that only mildly impact the catalytic activity, namely T520I (Haack et al, 2013), F154L (Haack et al, 2013), and G132R (Paucar et al, 2018). Mutations are shown in dark and light purple for the NTD and the CTD of ELAC2, respectively. The rest of ELAC2 is in gray, and the tRNA is in red. (**C**) Cleavage activity of the ELAC2 mutants on $tRNA^{His}$-CCA assessed by TBE-UREA PAGE. None of the mutations significantly affects the 3′-CCA antideterminant effect. The WT panel is the same as in Fig. 5A, shown here for an easier side-to-side comparison. Representative gel images of technical triplicates and biological duplicates for WT. Source data are available online for this figure.

 © The Author(s)
